# Awareness of Care Managers Concerning Grief Care for Older Bereaved Individuals Living Alone Following the Loss of Their Spouse: A Qualitative Research †

**DOI:** 10.3390/nursrep15100346

**Published:** 2025-09-24

**Authors:** Kazumi Hirano

**Affiliations:** Department of Nursing, Faculty of Health Care and Medical Sports, Teikyo Heisei University, 4-1 Uruido Minami, Ichihara-City 290-0193, Chiba, Japan; kazumi.hirano@thu.ac.jp; Tel.: +81-090-1841-4318

**Keywords:** grief care, end-of-life, households of older couples, older bereaved, older individuals living alone, nursing care support specialists

## Abstract

**Background/Objectives**: Japan has the most rapidly aging population worldwide, with older adults expected to reach 38.7% by 2070. Furthermore, individuals ≥ 60 years old desire to spend their final days at home. Following the establishment of the Long-Term Care Insurance System in 2000, care managers at home care support facilities continually assist most older adults receiving home care. Moreover, home-based end-of-life care is expected to increase. Therefore, the care manager’s role will be crucial in providing end-of-life care support and offering grief care to bereaved families following a patient’s passing. In this study, we aimed to clarify the perceptions of care managers regarding grief care for older bereaved individuals following the loss of their spouse and living alone. **Methods**: Seventeen care managers with prior experience in grief care for older bereaved individuals who became independent after the death of their spouse were interviewed. The 17 care managers comprised 2 men and 15 women, aged between their late 30s and early 60s. Qualitative data analysis was performed. **Results**: The following five categories were generated regarding the perceptions of care managers: necessity of supporting older bereaved individuals, ambivalent feelings towards grief care, death considerations, need for reflection, and challenges in implementing grief care. **Conclusions**: Care managers recognized the importance of maintaining a continued relationship before death, including the need for assessing older bereaved individuals, and collaborating with multiple professions in grief care.

## 1. Introduction

Japan has the most rapidly aging population, with its aging rate anticipated to reach 38.7% by 2070 [1]. Households with individuals aged ≥ 65 years account for 50.6% of all households, while those consisting solely of couples and single-person households account for approximately 30%. In Japan, it is estimated that by 2050, 26.1% of men and 29.3% of women aged ≥ 65 years will live alone [2]. In total, 51% of those aged ≥ 60 years answered that they wanted to spend their final moments at ‘home’ [3]. As Japan works to improve its home healthcare system [4], expectedly more people will wish to receive home-based care until the end, leading to an increase in home-based end-of-life care, which may require grief care for the bereaved.

A survey targeting bereaved families revealed that stress in various aspects of life is greatest following the death of a spouse [5]. Furthermore, bereavement has physical and mental effects on survivors [6,7]. Hongo et al. [8] reported that bereaved families needed comfort and encouragement, usually up to the 49th day after death. Approximately 10% of people needed this support until around the first anniversary. Concerning feelings of loneliness after loss, reportedly, loneliness is strongest within the first year after death and gradually diminishes over time [9]. Furthermore, conflicted feelings regarding bereavement from a spouse or family member often continue after a long period [10]. Moreover, family members are unable to consult anyone about the pain of loss [11]. Hirano [12] reported that older bereaved individuals who cared for their spouses at home and are currently living alone desire to connect with professionals with whom they can discuss their feelings of loneliness and anxiety. However, issues such as the importance of relationships with supporters have been reported [13,14].

Regarding the necessity of support for bereaved families, it is clearly stated in the WHO’s “Definition of Palliative Care” [15] that “7 Palliative care, offers a support system to help the family cope during the patient’s illness and in their bereavement” and “8 Palliative care uses a team approach to address the needs of patients and their families, including bereavement counseling, if indicated.” In the United States and the United Kingdom, support for bereaved families is often covered by insurance [16,17], and systems such as charities for the bereaved have been established [18]. Moreover, the services widely provided range, including information related to support, assistance with daily life difficulties, counseling, and more [19]. Although there is no public support in Japan, the bereavement care guidelines [20] were published in 2022, indicating the growing need for bereavement support initiatives.

Stroebe & Stroebe [21] defined grief as “an emotional response to loss, including various psychological and physical symptoms.” Sakaguchi [22] stated, “Many of the emotions, physical symptoms, and problem behaviors that arise from significant loss, including bereavement, are temporary and normal reactions that anyone can experience.” Grief and reactions vary across individuals [23]. Furthermore, grief changes over time, with its expression showing cultural differences [24]. Losing something important can cause varying emotions, including anger and guilt [25]. Many grief reactions are normal and subside naturally, often without necessary professional intervention [20].

However, bereavement is among life’s greatest stressors, affecting the physical and mental health of bereaved family members in several ways. These can cause worsening physical illnesses, increased mortality, suicide rates, and increased prevalence of depression, thereby disrupting daily life [26,27]. If grief persists for an extended time due to the pain of bereavement being carried alone, it may develop into complicated grief, requiring specialized care and treatment. Furthermore, determining that support is needed for the bereaved may be possible if there are others to consult or professionals involved before the bereavement. Therefore, complicated grief can be prevented when provided with appropriate support.

Care for a person is based on dignity, respecting their existence and value, and caring for them while preserving their humanity and enabling them to live their lives in a way that is true to themselves. Person-centered care, which fully considers the individuality and human dignity of those seeking care, is essential to quality care [28]. It enables meeting and addressing the physical, psychological, psychosocial, and spiritual needs of older adults [29]. Family, relatives, and friends are the most common sources of support for grieving individuals, and the most commonly sought support was a combination of emotional and practical support [30]. Grief care involves caregivers providing emotional support to grieving individuals and helping them overcome their pain [31]. Furthermore, demonstrating empathy through concrete actions is important, alongside emotional support [22,25,32]. Yamamoto [33] defines grief care as “care provided by supporters to help individuals cope with grief and bring about some degree of healing.” Grief care is intended to help alleviate the deep sadness and pain caused by the death of a loved one [34]. Grief care should help grieving individuals accept their emotions, rebuild their relationship with the deceased, and adapt to a new lifestyle [35,36]. Sakaguchi [22] states, “The most fundamental aspect of grief care is to respect the other person’s feelings and gently empathize with them.” Therefore, care managers should utilize interpersonal support techniques to support bereaved families while encouraging them to express their emotions.

Following the establishment of Japan’s long-term care insurance system in 2000, care managers from home care support offices have supported most older patients receiving home care. Therefore, they will continually play an important role in end-of-life care support and in grief care for bereaved families after the patient’s passing. According to Hirano [37], care managers have stated that they provide some form of support when they determine that continuous support is needed for the bereaved family, even after the patient’s passing and the contract has ended. However, studies that have focused on support for older bereaved individuals who have become sole residents after the death of a spouse in the households of older couples supported by care managers are lacking. Therefore, in this study, we aimed to clarify the perceptions of care managers regarding grief care for older bereaved individuals who have lost their spouse and have been living alone.

## 2. Materials and Methods

### 2.1. Research Design

Although the need for bereavement support is increasing in Japan, public support systems are still underdeveloped compared to other countries.

In this study, we adopted a qualitative cross-sectional research design using semi-structured interviews to explore care managers’ perceptions of grief care for elderly bereaved individuals living alone. The qualitative approach provides detailed insights into care managers’ practices regarding grief care for older adult bereaved individuals who have lost their spouses and are living alone. This methodology was adopted to reveal complex aspects that cannot be fully captured by quantitative methods.

### 2.2. Research Participants

The service areas of the care managers surveyed are the municipalities where their offices are located and neighboring municipalities. Each care manager was responsible for approximately 35–40 users. This region, like the rest of Japan, has an aging population. This study focused on 17 certified care managers with full-time engagement at home care support service offices for over three years, who had experienced practicing grief care for older bereaved individuals who became lonely after the loss of their spouses. We used the Benner’s [38] criteria for full competence for the selection criteria of the care managers, assuming at least three years of experience. Care managers with <3 years of experience and with no experience practicing grief care for older bereaved families who have lost their spouse and are lonely were excluded from the selection process. Participants who met specific criteria related to the research objectives were selected using convenience sampling (snowball sampling).

### 2.3. Data Collection Methods

The survey was conducted between 9 June 2020, and 2 July 2020. Each participant underwent one semi-structured interview. The interviews were conducted face-to-face in a private room selected by the participants to protect privacy. The average interview time was 72 min per person (range: 56–100 min). In addition, permissions were obtained for recording the interviews. Participants were asked about their basic attributes (age, gender, basic qualifications, and years of experience as a care manager) and the timing and frequency of visits to the bereaved families. Furthermore, they were asked to freely vocally express the emotions they experienced while providing grief care to older adult bereaved families who had lost their spouses and were living alone. A specific target for the number of interviews was not set; rather, there was continuous interviewing until data saturation was reached. Regular discussions were held with research collaborators after each interview to confirm saturation. Therefore, saturation was reached at the 17th interview since no new categories were generated after the 15th interview.

### 2.4. Analysis Method

Based on Sato’s [39] qualitative data analysis method, interview recordings were transcribed verbatim and divided into the smallest paragraphs possible for their interpretation. These were used as the unit of analysis. Next, coding was performed while focusing on the awareness of care management professionals regarding grief care for older adult bereaved families living alone after the death of their spouse. Categories were generated by identifying commonalities among the codes and an increase in the abstraction level. Category generation involved repeated phases of reviewing characteristics and naming of categories while returning to the data and codes. Furthermore, the relationships between the categories were explored by comparing their similarities and differences, and structuring was attempted. The results were shared with participants who consented to the analysis at the final category generation stage. In addition, the accuracy of the analysis results was confirmed.

### 2.5. Ensuring Rigor

The reliability of this study was confirmed using Lincoln and Guba’s [40] reliability assessment criteria. The authors have work experience as nurses and care managers. In addition, the sociologist who collaborated on this study has a PhD in sociology and is an important scholar who contributed to the establishment of Japan’s long-term care insurance system. He is familiar with Japan’s long-term care insurance system and the role of care managers. Furthermore, he critically examined the awareness of care managers and their role in Japan’s long-term care insurance system throughout the research process. We included a researcher with experience in qualitative research and as a care manager. Continuous discussions were conducted to address emerging biases and maintain a care manager-centered perspective. We regularly sought advice, received feedback on data collection, analysis, and outcome evaluation throughout the research process, and endeavored to clarify the research process. These processes were designed to enhance the reliability and credibility of the research findings.

### 2.6. Ethical Considerations

This study was approved by the Research Ethics Review Committee of Chiba University of Science (Approval number: R01-16, 31 March 2020). Verbal explanation was provided to the research participants using an explanatory document about the purpose and content of the study, and that the research participation was voluntary. Withdrawing would not place them at a disadvantage, ensuring privacy protection, strict maintenance of anonymity, and securely handling the data and personal information, including the content obtained, so as to not be used for purposes other than this study. Informed consent was obtained from all participants.

## 3. Results

### 3.1. Overview of Research Participants (Table 1)

Seventeen care managers co-operated with the study. There were 2 males and 15 females, with their ages ranging from the late 30s to the early 60s. The basic qualifications included 15 welfare professionals and two medical professionals, with an average of 10.5 years of experience as care managers. Bereavement visits, usually one or twice, were conducted within the first-year post-loss, with most occurring within one month and around the 49th day mark.

### 3.2. Care Managers’ Perceptions of Grief Care for Older People Living Alone (Table 2)

With respect to care managers’ perceptions of grief care for older bereaved individuals who have become solitary owing to the loss of a spouse, five categories were generated: necessity of supporting older bereaved individuals, ambivalent feelings towards grief care, considerations of death, need for reflection, and challenges in implementing grief care. These were further divided into 11 subcategories. The category is represented by [ ], subcategory as < >, part of the code as ‘ ’, and sections that include additional interpretations related to the content are indicated as ( ). Note that ( ) in Table 2 indicates the number assigned to the code.

**Table 2:** Care managers’ perceptions of grief care for older people living alone.

Category	Subcategory	Code
The necessity of supporting older widows and widowers	The importance of relationships with older bereaved families	Just because the same words are spoken to different people, it doesn’t mean they’ll be received in the same way. I believe that the way we reach out depends on our relationship (F8). I think that the ability to enter without hesitation is influenced by prior interactions (K3). The frequency of visits and the degree of engagement change depending on the depth of the relationship (E11).
	The necessity of assessing older bereaved individuals.	I am forced to consider the response. Each person’s situation, life, and relationships are different, and assessment is important (O9). It is difficult to judge and evaluate the level of support needed (Q18). I question whether I was able to view not only the users but also their surviving spouses comprehensively (K4).
	The need for multidisciplinary collaboration	By entrusting areas of expertise and specialties to others, I can receive support in areas where I lack knowledge (C14). If we can connect with the same mindset, we can collaborate closely. I feel that exchanging information, even about trivial matters, is important (L18).
Ambivalent Emotions in Grief Care	The pain of accepting feelings	There are times when it can be painful, as it may serve as a release for stress and feelings (G7). Although all I can do is listen, I do not want to listen superficially. There are also times when I hurt myself (H2).
	The sense of security for older bereaved individuals	I realize that after visiting the older bereaved family, having tea and talking with them, I feel relieved (D9). I think it is good for those who have family support (D14). It also becomes a care for my own heart (C11).
Consideration of Death	Consideration of Death	I am made to think about death (A2). I am prompted to consider what is necessary to fulfill how I want to face the end (D4). It is important to talk about death from a time when one is healthy (P27).
	Loss of Relationship	I feel a sharp sensation of our relationship suddenly severing (N5). The contract has ended, but when I return to the site from that time, there is an indescribable feeling (M15). I can feel the time that will not return (I11).
The Necessity of Reflection	The need to reflect on grief care	By reflecting on the grief care I have provided, there are times when I feel that what I have done was good, but there are not many opportunities to do so (D29). I feel the need for a space where I can reflect and share my own story (B8). Looking back, I realize that what I did not think of as grief care was actually grief care (C12).
	The need to share grief care	I feel that if we can share our experiences with each other, the practice of grief care will progress, supporting the bereaved in their reintegration into society and preventing losses to society (C20). I hope that by sharing this experience, it will become something transformative (N10). If there is an opportunity to leverage this experience and listen to the stories of others, I would like to try (H9).
Challenges in Implementing Grief Care	The necessity of grief care education	I feel that as home care for the terminally ill increases, there is a growing need for knowledge and skills related to grief, but there are no opportunities to learn (D32). Since I have not received education in grief care, I am struggling with it (F9). My grief care is self-taught, and I lack confidence, so I want to study it properly (D21).
	The necessity of support systems for older bereaved individuals	I believe that if there is a support system in the community, we can provide continuous support (J7). For bereaved families who cannot accept their loss, I feel it is important to support them in finding a new life and joy, but I struggle with who should take on that role (B1). I think it is concerning to leave those who are still grieving alone, but I am troubled that I cannot visit them frequently (H4).

#### 3.2.1. The Necessity of Supporting Older Survivors

It was composed of three subcategories: <The importance of relationships with older bereaved families>, <The necessity of assessing older bereaved individuals>, and <The need for multidisciplinary collaboration>.

As stated in the comments, ‘Just because the same words are spoken to different people, it does not mean they will be received in the same way. I believe that the way we reach out depends on our relationship (F8).’ ‘I think that the ability to enter without hesitation is influenced by prior interactions (K3). ‘This highlights the importance of these relationships, even during the healing process. Furthermore, I felt the necessity of assessing the remaining older bereaved, as ‘we have no choice but to consider how to respond. Each person’s situation, lifestyle, and relationships are different, so assessment is important (O9),’ and ‘It is difficult to judge and evaluate the level of support needed (Q18).’ Conversely, the recognition that ‘by entrusting one’s areas of expertise or skills to others, support can be received in areas where knowledge is lacking (C14).’ ‘ If we can connect with the same mindset, we can collaborate closely. I feel that exchanging information, even about trivial matters, is important (L18).’

#### 3.2.2. Ambivalent Emotions in Grief Care

It was composed of two subcategories: <The pain of accepting feelings> and <The sense of security for older bereaved individuals>

Care managers have experienced hardships, such as ‘there are times when it can be painful, as it may serve as a release for stress and feelings (G7),’ while interacting with older bereaved individuals. However, ‘I realize that after visiting the older bereaved family, having tea and talking with them, I feel relieved (D9),’ ‘It also becomes a care for my own heart (C11). ‘The care support specialist found comfort in the unexpectedly lively older bereaved.

#### 3.2.3. Consideration of Death

It consists of two subcategories: <Consideration of Death> and <Loss of Relationships>, and through grief care, many narratives led to thinking about death.

In addition, they recognized that ‘I am prompted to consider what is necessary to fulfill how I want to face the end (D4),’ and ‘It is important to talk about death from a time when one is healthy (P27).’ Furthermore, respondents described a sense of loss, as though relationships they had built up over the years had been suddenly severed, with comments such as ‘I feel a sharp sensation of our relationship suddenly severing (N5) ‘ and ‘I can feel the time that will not return (I11).’

#### 3.2.4. The Necessity of Reflection

It was composed of two subcategories: <The need to reflect on grief care> and <The need to share grief care>.

Responses such as ‘By reflecting on the grief care I have provided, there are times when I feel that what I have done was good, but there are not many opportunities to do so (D29)’ and ‘ I feel the need for a space where I can reflect and share my own story (B8)’ indicated a recognition of the necessity for opportunities for reflection. Furthermore, responses such as ‘I feel that if we can share our experiences with each other, the practice of grief care will progress, supporting the bereaved in their reintegration into society and preventing losses to society (C20)’ and ‘I hope that by sharing this experience, it will become something transformative (N10)’ emphasized the necessity in sharing everyone’s experiences through reflection.

#### 3.2.5. Challenges in Implementing Grief Care

It was composed of two subcategories: <The necessity of grief care education> and <The necessity of support systems for older bereaved individuals>.

‘I feel that as home care for the terminally ill increases, there is a growing need for knowledge and skills related to grief, but there are no opportunities to learn (D32).’ Since I have not received education in grief care, I am struggling with it (F9) ‘’My grief care is self-taught, and I lack confidence, so I want to study it properly (D21).’ From these comments, due to the increasing need for home-based end-of-life care and the recognition that knowledge and skills related to grief are essential, there is a growing desire for grief care training, particularly in anticipation of an aging society. Furthermore, the statement, ‘I believe that if there is a support system in the community, we can provide continuous support (J7)’ and ‘For bereaved families who cannot accept their loss, I feel it is important to support them in finding a new life and joy, but I struggle with who should take on that role(B1)’ demonstrated that a support system within the community would enable uninterrupted support for bereaved families with older adults who continue to grieve. This highlighted the necessity for a system to support bereaved families within the community.

## 4. Discussion

### 4.1. Necessity to Support Older Widows and Widowers

Care for bereaved families is important in maintaining the continuity of the relationship between the bereaved and their supporters before the loss [41]; as it is said, ‘It is a voice of concern arising from the relationship,’ which existed in the extension of care given while they were alive. For older individuals who are left alone after the loss of their spouse, receiving continuous support from care managers who understand their situation can reduce regret and alleviate the pain following their loss [42], which is considered meaningful. Furthermore, as reported by Okamoto & Hiramatsu [43], it is important to provide early support so that caregivers and families can discuss the patients’ feelings before bereavement. Caregivers who have been providing support before the loss are aware of the thoughts and concerns of the patients and their families, making them trustworthy and reliable helpers for the bereaved [44]. Furthermore, they are important in grief care.

Hirano [37] reported that it is important for care managers to assess older bereaved individuals, as it helps establish a continuous support system from medical care to aftercare. Older bereaved individuals living alone often become lonely, and it is particularly difficult for them to seek mental support or start new activities. In addition, they may encounter challenges related to their physical strength and health. Furthermore, older adult bereaved family members may be unaware of their own needs, do not seek support, and may not receive the care they need [45]. Therefore, care managers must recognize the necessity of bereavement assessments. Care managers can assess the situation of older bereaved individuals who are concerned during interactions, namely, by confirming their circumstances after death, allowing them to determine the necessity of ongoing support. However, the method of conducting assessments is left to individual care managers, and the current situation has not been established. Therefore, establishing a method to assess family caregivers is challenging.

Furuse [46] reported that care managers with basic qualifications who are non-medical professionals are aware of their reduced medical knowledge and take actions to directly consult experts when medical knowledge or judgment is necessary. In this study, there is a strong recognition of the necessity to collaborate with multiple professions, as many certified care managers have a non-medical basic qualification. There were many narratives indicating that “by relying on the specializations and areas of expertise of others, I can receive support for things I do not possess.” Furthermore, Yanagihara [47] reported that, according to basic qualifications, awareness of one’s own effectiveness and limitations as a care manager should lead to the management of services that address those limitations. The care managers targeted in this study should be aware of their strengths and weaknesses and recognize the necessity of interdisciplinary collaboration in grief care. Therefore, to support the home care team for older couples, it can be inferred that they recognized the need to select team members suitable for them [48] and continued to be the central support for the older bereaved after the patient’s passing.

### 4.2. Ambivalent Feelings Embraced in Grief Care

There is a narrative that ‘stress can serve as an emotional outlet, and it can be painful. Bereaved families often have unmet needs, such as communication and practical support [49], and the stress they feel is sometimes directed at care managers.’ However, support for the pain in older bereaved individuals may prevent complex grief [50]. Moreover, it can be assumed that while facing their own difficulties, care managers practiced grief care tentatively, thereby fulfilling their professional responsibilities. Moreover, as it was mentioned, ‘I realized that I, who had visited out of worry, returned after having tea and talking, feeling relieved,’ indicating awareness of these conflicting emotions.

### 4.3. Consideration of Death

Views on life and death are considered important in end-of-life care provided by certified care managers [51]. Supporting individuals in receiving an acceptable “end of life” is one of the areas of expertise required of care managers [52]. The background behind the Japanese perception of death is centered on family-centeredness, indicating the importance of supporting the family as a secondary patient [53]. Care management professionals who provide support alongside family members cultivate perspectives on life and death through care and interactions [54]. However, care support specialists often grapple with fear and feelings of powerlessness regarding death during end-of-life support [55]. In addition, they need to be aware of their experiences of loss and mortality, reflect on their own lives and deaths individually, and construct their perspectives on life and death [56].

Furthermore, because the contract ends with the death of the patient, their relationship with their family naturally ends. However, there are instances in which caregiver support specialists developed a sense of familial connection during their involvement [55]. Care managers view families as a unit and place emphasis on caring for bereaved families [57]. They may have experienced a secondary feeling of loss regarding relationships built with older bereaved individuals, suggesting an awareness of the considerations surrounding death.

### 4.4. Necessity for Reflection

Care managers wanted to reflect on the support they had provided. Supporting the bereaved is often left to the individual’s responsibility [31]. Among many care support specialists struggling with grief care, intentionally expressing emotions and undertaking the work of rebuilding oneself [55] serves as a space where they can disclose their concerns and regrets about grief care, which may also lead to a reduction in their mental burden. In addition, it can be considered that there was a recognition of the necessity for a place where members could affirm their learning and support by obtaining practical content and opinions from one another. Furthermore, care managers sought the dissemination of grief care practices, recognizing them as a meaningful contribution to society.

Furthermore, care managers recognized the need to share grief care. Care managers, reflecting on the care they have provided, provide an opportunity for learning, leading to improved quality of care [58]. Sharing the content and knowledge of grief care practiced by each care manager within the community and workplace promotes awareness and new learning, leading to improved quality of care. By sharing their practices, they recognized the necessity of grief care and recognized the spread of grief care practices as a meaningful contribution to society, and worked to promote its spread.

### 4.5. Challenges in Implementing Grief Care

According to Hirano [37], care managers implement grief care; however, they report struggling with the inability to provide a clear meaning. Older people living alone often experience emotional loneliness and social isolation [59,60]. It has been reported that the risk of suicide increases within one year of the death of a loved one [61]; therefore, providing appropriate support before and after bereavement is important. Grief care requires consideration when bereaved individuals are allowed to express their emotions [12]. However, many care managers do not have nursing qualifications and are so-called non-medical care managers who have received little education on discussing death and end-of-life issues with older individuals and their families [62]. Regarding training on grief care, studies concerning care managers are lacking; however, there are reports stating that among the challenges faced by home care nurses, there are few opportunities to learn about grief care [43,63]. Reports show that only about 30% of home care agencies provide education on grief care [64]. Although training related to end-of-life care is included in the legally mandated training for care managers, there is no training content on grief care. By participating in grief care training, it is believed that one’s understanding of grief care will deepen.

Moreover, while the care manager is engaged in the mourning process following a client’s death, they must continue to support the other client. Many have expressed, ‘I worry that those who are still grieving should not be left alone; however, I struggle because I cannot visit frequently.’ Visiting bereaved families is not established as part of the system and time constraints are involved; therefore, providing ongoing support is difficult. In addition, there is no remuneration for bereavement support, as it is not covered by health insurance [12,34]. We found numerous narratives wishing for a support system for older bereaved individuals in the community, expressing sentiments such as, ‘If there were a support system in the community, I think continuous support could be provided.’ There is anticipation that older individuals who become bereaved and live alone will internalize their grief of losing a loved one. However, if community support increases, it may lead to the prevention of issues such as social withdrawal, depression, and complicated grief. It is believed that, in Japan, to provide more appropriate support to those who need assistance after losing a loved one, it is necessary to consider establishing support systems for bereaved families.

### 4.6. Limitations and Future Challenges of This Study

The 17 study participants were care managers from a limited area, which may not represent the general characteristics of care managers in Japan. Therefore, there are limitations in generalizing this study’s results. Future studies should aim to increase the number of participants and understand the actual conditions of care support specialists working in various regions, considering regional characteristics. In addition, the recruitment from a limited area may have caused selection bias.

Nevertheless, this study provides important insights into the challenges recognized by care managers providing grief care to bereaved families.

## 5. Conclusions

Japan is expected to see an increase in home-based end-of-life care in the future. Furthermore, care managers are expected to continue playing an important role in providing end-of-life care and in providing grief care to bereaved families after the patient’s death.

We discovered that care managers’ perceptions of grief care for older adult bereaved families include the need to support bereaved families with older adults who live alone after offering home-based care to their spouse. In addition, awareness of ambivalent feelings associated with grief care, consideration for death, the need for self-reflection, and challenges in providing grief care.

Care managers recognized the importance of maintaining ongoing relationships with older adult who become isolated following the death of their partner. Furthermore, they recognized the need for assessing older adult bereaved families and the importance of multidisciplinary collaboration in grief care. They valued connections with bereaved families and sought opportunities to reflect on their own grief despite facing difficulties. However, there is insufficient knowledge of grief care and systems to support older adult bereaved families in the community. Our findings underscore the need for further education on grief care for care managers. Furthermore, the findings suggest the need to establish a system for supporting older adult bereaved family members in the local community.

## Figures and Tables

**Table 1:** Overview of the research participants.

Research Participants	Sex	Age	Basic Job Categories	Years of Experience as a Care Manager	Main Periods for Visiting Bereaved Families	Number of Visits to the Bereaved Family
A	Woman	In their 50 s	Caregiver	8 years	Shortly after passing away, around the forty-ninth day.	1–2
B	Woman	In their 60 s	Caregiver	10 years and 4 months	Immediately after passing away, first anniversary of the death	2–3
C	Woman	In their 60 s	Medical profession	3 years and 3 months	Shortly after passing away, around the forty-ninth day.	2–3
D	Woman	In their 60 s	Caregiver	17 years and 1 month	Immediately after passing away, around one month later.	1–2
E	Woman	In their 60 s	Caregiver	20 years and 2 months	Immediately after passing away—the first seven days	1–2
F	Woman	In their 60 s	Caregiver	10 years	Shortly after passing away, around the forty-ninth day.	1–2
G	Woman	In their 60 s	Caregiver	11 years	Immediately after passing away—the first seven days	1
H	Woman	In their 30 s	Caregiver	7 years and 2 months	Around the forty-ninth day	1
I	Woman	In their 50 s	Caregiver	10 years and 2 months	Around the forty-ninth day	1
J	Man	In their 40 s	Caregiver	12 years	Around the forty-ninth day	1
K	Woman	In their 40 s	Caregiver	8 years and 4 months	Immediately after passing away	1
L	Woman	In their 50 s	Caregiver	6 years and 1 month	Around the forty-ninth day	1
M	Woman	In their 50 s	Caregiver	11 years and 6 months	Immediately after passing away—the first seven days	1
N	Woman	In their 60 s	Caregiver	12 years	Immediately after passing away	1
O	Woman	In their 50 s	Medical profession	20 years	Shortly after passing away, around the forty-ninth day.	1–2
P	Man	In their 40 s	Caregiver	3 years and 11 months	The first seven days after death	1
Q	Woman	In their 50 s	Caregiver	13 years and 1 month	Shortly after passing away, around the forty-ninth day.	2

## Data Availability

The raw data supporting the conclusions of this article will be made available by the authors on request.

## Public Involvement Statement

No public involvement in any aspect of this research.

## Guidelines and Standards Statement

This manuscript was drafted in accordance with the Standards for Reporting Qualitative Research [65].

## References

[1] Cabinet Office (2024). Version of the White Paper on Aging Society. https://www8.cao.go.jp/kourei/whitepaper/w-2024/zenbun/pdf/1s1s_01.pdf.

[2] Cabinet Office (2024). Edition of the Aging Society White Paper. https://www8.cao.go.jp/kourei/whitepaper/w-2024/zenbun/pdf/1s1s_03.pdf.

[3] Cabinet Office (2019). Edition of the Aging Society White Paper. https://www8.cao.go.jp/kourei/whitepaper/w-2019/html/zenbun/s1_3_1_4.html.

[4] (2023). Ministry of Health, Labour and Welfare, Regarding the Establishment of Home Medical Care Systems. https://www.mhlw.go.jp/content/10800000/001146149.pdf.

[5] Holmes T.H., Rahe R.H. (1967). The social readjustment rating scale. J. Psychosom. Res..

[6] Stroebe M., Schut H., Stroebe W. (2007). Health outcomes of bereavement. Lancet.

[7] Cole M.G., Dendukuri N. (2003). Risk factors for depression among elderly community subjects: A systematic review and meta-analysis. Am. J. Psychiatry.

[8] Hongo S., Kondo K., Makino T., Kuze J., Higuchi K., Sugimoto H., Miyata K. (2003). Support needed by caregivers in terminal care for home-dwelling elderly—A survey targeting bereaved families. Term. Care.

[9] Suzuki H., Takigawa S. (2005). Loneliness and related factors in men who have experienced the loss of a spouse. Hosp. Care Home Care.

[10] Sugimoto T., Imai K.K. (2004). Analysis of the feelings of home-dwelling elderly people who have lost a spouse. Nurs. J. Kagawa Med. Univ..

[11] Onishi H., Hirayama M. (2010). Clinical Studies in Death and Life Series 2, What Has Emerged from the Bereaved Outpatient Clinic. To Recover from the Grief of Loss.

[12] Hirano K. (2023). The role of home care nurses for elderly individuals who have provided care for their spouse at home: Insights from the narratives of elderly individuals living alone. Hosp. Care Home Care.

[13] Chang E., Bidewell J., Hancock K., Johnson A., Easterbrook S. (2012). Community palliative care nurse experiences and perceptions of follow-up bereavement support visits to carers. Int. J. Nurs. Pract..

[14] Brownhill S., Chang E., Bidewell J., Johnson A. (2013). A decision model for community nurses providing bereavement care. Br. J. Community Nurs..

[15] World Health Organization (WHO) (2020). Palliative Care.

[16] National Health Service Mental Health. Feelings, Symptoms and Behaviours. https://www.nhs.uk/mental-health/feelings-symptoms-behaviours/.

[17] National Hospice and Palliative Care Organization Getting Grief and Loss Support. https://www.caringinfo.org/planning/grief-and-loss/getting-grief-and-loss-support/.

[18] Cruse Bereavement Support. Get Support. One to One Support. https://www.cruse.org.uk/get-support/one-to-one/.

[19] Ghesquiere A., Bagaajav A., Metzendorf M., Bookbinder M., Gardner D.S. (2019). Hospice bereavement service delivery to family members and friends with bereavement-related mental health symptoms. Am. J. Hosp. Palliat. Care.

[20] Japanese Psycho-Oncology Society, Japan Society of Cancer Supportive Care (2022). Bereavement Care Guidelines.

[21] Stroebe W., Stroebe M.S. (1987). Bereavement and Health: The Psychological and Physical Consequences of Partner Loss.

[22] Sakaguchi Y. (2023). Grief Care for Those Who Have Lost a Loved One.

[23] Seto N., Hirose H.T. (2023). Grief Care and Grief Counseling: A Practical Guide to Supporting Bereavement and Grief.

[24] Sakaguchi Y. (2022). Introduction to Grief Studies—Learning about the Grief of Losing a Loved One.

[25] Takagi K., Akimoto T. (2023). A Must-Read for Those Involved in Grief Care and Spiritual Care.

[26] Onisi H., Ishida M. (2014). Family and Bereavement Care. Jpn. Soc. Psychosom. Med..

[27] Brodbeck J., Jacinto S., Gouveia A., Mendonça N., Madörin S., Brandl L., Schokking L., Rodrigues A.M., Gonçalves J., Mooser B. (2022). A web-based self-help intervention for coping with the loss of a partner: Protocol for randomized controlled trials in 3 countries. JMIR Res. Protoc..

[28] Berntsen G.R., Yaron S., Chetty M., Canfield C., Ako-Egbe L., Phan P., Curran C., Castro I. (2021). Person-centered care (PCC): The people’s perspective. Int. J. Qual. Health Care.

[29] Sillner A.Y., Madrigal C., Behrens L. (2021). Person-centered gerontological nursing: An overview across care settings. J. Gerontol. Nurs..

[30] Benkel I., Skoglund J., Enstedt D., Hård af Segerstad Y., Öhlén J., Nyblom S. (2024). Understanding the needs for support and coping strategies in grief following the loss of a significant other: Insights from a cross-sectional survey in Sweden. Palliat. Care Soc. Pract..

[31] Riguzzi M., Thaqi Q., Lorch A., Blum D., Peng-Keller S., Naef R. (2024). Contextual determinants of guideline-based family support during end-of-life cancer care and subsequent bereavement care: A cross-sectional survey of registered nurses. Eur. J. Oncol. Nurs..

[32] Louise I.R., Nikki Y.Y., Yasmine A.A., Laurie S., Mark E.F., Adam M.D. (2023). The impact of bereavement support on psychological distress in family members: A systematic review and meta-analysis. Crit. Care Resusc..

[33] Yamamoto T. (2014). Clinical Psychology of Loss and Grief: Modal Model and Morning Work.

[34] Riguzzi M., Thaqi Q., Peng-Keller S., Lorch A., Blum D., Naef R. (2024). Adoption of evidence-based end- of- life and bereavement support to families in cancer care: A contextual analysis study with health professionals. J. Clin. Nurs..

[35] Niino K., Patapoff M.A., Mausbach B.T., Liu H., Moore A.A., Han B.H., Palmer B.W., Jester D.J. (2025). Development of loneliness and social isolation after spousal loss: A systematic review of longitudinal studies on widowhood. J. Am. Geriatr. Soc..

[36] Nazlıer E.N.Y., Özkan Y. (2024). An Examination of the Factors Influencing Grief Cognition and Meaning Reconstruction among Older Bereaved spouses. Death Stud..

[37] Hirano K. (2023). Methods of grief care implemented by care managers—Targeting solitary elderly individuals who have lost their spouses. J. Care Manag..

[38] Banner P., Wrubel J.T., Nanba T. (1999). The Primacy of Caring: Stress and Coping in Health and Illness.

[39] Sato I. (2008). Qualitative Data Analysis—Principles, Methods, Practice.

[40] Lincoln Y.S., Guba E.G. (1985). Naturalistic Inquiry.

[41] Aoun S.M., Rumbold B., Howting D., Bolleter A., Breen L.J. (2017). Bereavement support for family caregivers: The gap between guidelines and practice in palliative care. PLoS ONE.

[42] Holtslander L., Baxter S., Mills K., Bocking S., Dadgostari T., Duggleby W., Duncan V., Hudson P., Ogunkorode A., Peacock S. (2017). Honoring the voices of bereaved caregivers: A Metasummary of qualitative research. BMC Palliat. Care.

[43] Okamoto F., Hiramatsu M. (2018). Difficulties faced by home care nurses in grief care for families of terminally ill cancer patients at home. J. Jpn. Home Care.

[44] Sakaguchi Y. (2021). Death and grief care in a super-aging society. J. Gerontol. Nurs..

[45] Pragmatic RCT to Assess the Effectiveness of an Online Self-Help Programme for Older Adults After Spousal Bereavement. https://clinicaltrials.gov/study/NCT05156346.

[46] Furuse M. (2017). The support process for terminal cancer patients by skilled care managers with basic qualifications in welfare work. Hosp. Care Home Care.

[47] Yanagihara K. (2006). Analysis of the ‘awareness of end-of-life care’ among care support professionals and related factors. Bulletin of the Department of Health Sciences.

[48] Dōzono H., Okada S., Shirasawa M. (2007). The role of care management specialists in home terminal care for the elderly. Life Sci. Res..

[49] Holm M., Årestedt K., Öhlen J., Alvariza A. (2020). Variations in grief, anxiety, depression, and health among family caregivers before and after the death of a close person in the context of palliative home care. Death Stud..

[50] Redshaw S., Harrison K., Johnson A., Chang E. (2013). Community nurses’ perceptions of providing bereavement care. Int. J. Nurs. Pract..

[51] Shimanuki M., Matsui M. (2011). Care management and perspectives on life and death in hospice care for patients with terminal cancer by certified care managers. Hosp. Home Care.

[52] Mori S. (2015). Revisiting perspectives on life and death. Care Manag..

[53] Ogasawara C. (2018). End-of-Life Care Nursing—Fundamentals and Practice.

[54] Nagayama H., Ogasawara C., Tainaka Y. (2021). The structure of nurses’ views on life and death related to end-of-life care—Development and verification of a scale for nurses’ views on life and death. J. Jpn. Soc. Nurs. Sci..

[55] Taiga Y., Mori T. (2018). The process of grief work associated with the loss of the care manager’s role in home healthcare support—A study on support for elderly individuals living alone with cancer in view of end-of-life care. Bol. Aichi Prefectural Univ. Educ. Welf..

[56] Sugino M., Akiyama S. (2017). The perspective of non-medical professionals on life and death revealed from awareness of one’s own death—Using the life and death scale by Hirai et al. Hiroshima International University. J. Nurs..

[57] Thaqi Q., Riguzzi M., Blum D., Peng-Keller S., Lorch A., Naef R. (2024). End-of-life and bereavement support to families in cancer care: A cross-sectional survey with bereaved family members. BMC Health Serv. Res..

[58] Narimatsu J., Tanaka M., Tanaka A., Nagao N., Irino R., Torii J., Nomura M. (2024). End-of-life care management and views on life and death among care managers. J. Shikoku Assoc. Public Health.

[59] Van Baarsen B., Van Duijn M.A.J., Smit J.H., Snijders T.A.B., Knipscheer K.P.M. (2002). Patterns of adjustment to partner loss in old age: The widowhood adaptation longitudinal study. Omega.

[60] Cohen M.A. (2000). Bereavement groups with the elderly. J. Psychother. Indep. Pract..

[61] Erlangsen A., Jeune B., Bille-Brahe U., Vaupel J.W. (2004). Loss of partner and suicide risks among oldest old: A population-based register study. Age Ageing.

[62] Hirakawa Y. (2019). The role and challenges of care managers in advance care planning for the elderly. Hosp. Care Home Care.

[63] Mizobe Y., Matsugi K. (2020). Current status and challenges of grief care in home nursing: A literature review. Osaka Med. Coll. Nurs. Res..

[64] Watanabe A., Tomita S. (2021). Difficulties and educational issues faced by visiting nurses in grief care. J. Kawasaki Med. Welf. Soc..

[65] O’Brien B.C., Harris I.B., Beckman T.J., Reed D.A., Cook D.A. (2014). Standards for reporting qualitative research: A synthesis of recommendations. Acad. Med..

